# Root stomata in *Conium maculatum* (Apiaceae): anatomically verified occurrence and a comparative survey across Apioideae

**DOI:** 10.1093/aobpla/plag001

**Published:** 2026-02-10

**Authors:** Zahra Khazaei, Ali Bagheri, Dmitry Lyskov, Dörte Harpke, Frank R Blattner

**Affiliations:** Department of Plant and Animal Biology, Faculty of Biological Science and Technology, University of Isfahan, Hezar Jerib Street, Isfahan 81746-73441, Iran; Department of Plant and Animal Biology, Faculty of Biological Science and Technology, University of Isfahan, Hezar Jerib Street, Isfahan 81746-73441, Iran; Department of Higher Plants, Faculty of Biology, Lomonosov Moscow State University, 1–12 Leninskie Gory, Moscow 119234, Russia; Leibniz Institute of Plant Genetics and Crop Plant Research (IPK), Corrensstr. 3, Seeland-Gatersleben 06466, Germany; Leibniz Institute of Plant Genetics and Crop Plant Research (IPK), Corrensstr. 3, Seeland-Gatersleben 06466, Germany; German Centre of Integrative Biodiversity Research (iDiv) Halle-Jena-Leipzig, Puschstr. 4, Leipzig 04103, Germany; Form & Function

**Keywords:** *Conium maculatum*, ITS region, primary root anatomy, root stomata, *rps*16 plastid gene, subterranean epidermis

## Abstract

**Background and Aims:**

Stomata are specialized epidermal structures typically restricted to aerial organs of vascular plants. Their absence on roots has long been regarded as a general anatomical rule. Although rare reports in certain dicotyledonous taxa have described root stomata, these occurrences have been considered transient or developmentally anomalous. Within Apiaceae, no confirmed occurrence has previously been documented. This study aimed to investigate the anatomical presence of stomata on the primary roots of *Conium maculatum* L. seedlings.

**Methods:**

Seedlings of *C. maculatum* derived from wild-collected populations and genebank accessions were examined using light microscopy. Transverse sections and epidermal surface preparations were prepared to confirm root identity and epidermal features. Taxonomic identity was verified using morphological traits and molecular data (ITS and *rps16* sequences).

**Key Results:**

Morphologically distinct stomata with characteristic guard cells were observed on the primary root. Their distribution was sparse and irregular. Root identity was supported by the overall anatomical organization of the examined sections, including a uniseriate epidermis and a broad parenchymatous cortex. Stomatal complexes were consistently detected across all examined accessions of the species.

**Conclusions:**

This study provides the first anatomically verified and reproducible report of stomata on the primary root of *C. maculatum* within Apiaceae. These findings expand current knowledge of root epidermal anatomy in the family and indicate that further comparative surveys will be necessary to determine the taxonomic extent of this trait.

## Teaser

Stomata are generally considered absent from roots in vascular plants. In this study, we provide anatomically verified and reproducible evidence of stomata on the primary root of Conium maculatum. Using light microscopy and comparative screening across more than 30 Apiaceae taxa, we show that root stomata represent a consistent species-level anatomical feature in C. maculatum. These findings expand current understanding of root epidermal development in angiosperms.

## Introduction

Stomata are specialized epidermal structures that regulate gas exchange and transpiration in vascular plants. Under normal developmental conditions, they are restricted to aerial organs such as leaves, petioles, young stems, and reproductive parts, where they contribute to photosynthetic efficiency and water balance. Their absence from subterranean organs, particularly roots, has long been regarded as a fundamental anatomical trait, reflecting their nonphotosynthetic role and soil-embedded nature (Esau 1965, Fahn 1982). Although this general pattern holds for most angiosperms, a few isolated and taxonomically scattered reports have documented root-associated stomata under specific developmental or environmental conditions. In *Helianthus annuus*, stomata were described on seedling roots grown under moist-chamber conditions, occurring mainly in the upper part of the root-hair zone and displaying morphological irregularities indicative of limited or absent functionality (Tietz and Urbasch 1977). In *Pisum sativum*, stomata were observed only at the extreme root apex and were developmentally transient (Lefebvre 1985). Likewise, in *Pisum arvense* and *Ornithopus sativus*, stomata appeared sporadically within the elongation zone, where they were structurally irregular and notably unresponsive to abscisic acid, further suggesting a lack of functional capacity (Tarkowská and Wacowska 1988). In *Ceratonia siliqua*, mature stomata were reported in a defined apical root zone. Their guard cells lacked chloroplasts, persisting only as amyloplasts, and were therefore interpreted as nonfunctional (Christodoulakis et al. 2002). Collectively, these studies indicate that root stomata are rare, developmentally unstable, and generally nonfunctional anatomical anomalies rather than consistent traits. Within Apiaceae, a large and taxonomically complex family with more than 3700 species and over 400 genera worldwide (Downie et al. 2010, Magee et al. 2010, Clarkson et al. 2021), no occurrence of root stomata has been published. Despite extensive anatomical work on fruits, stems, and reproductive structures within the family, root epidermal characters remain almost entirely undocumented. Although molecular and phylogenetic studies have significantly advanced understanding of lineage relationships and trait evolution in the family (Ajani et al. 2008, Mousavi et al. 2020), anatomical investigations of subterranean organs are limited. Here, we report the first confirmed occurrence of structurally complete stomata on the primary root of poison hemlock, *Conium maculatum* L., a widely distributed toxic biennial herb in subfamily Apioideae. By examining seedlings derived from both wild populations and genebank accessions, we provide the first reproducible evidence of root-associated stomata in Apiaceae, describing their distribution, structural identity, and stability. These findings are interpreted in the context of previous anatomical reports of root stomata, providing the necessary comparative framework for evaluating their developmental origin and taxonomic distribution.

## Materials and methods

### Plant material and germination conditions

Seeds of *Conium maculatum* were sourced from two principal origins: (i) field-collected individuals obtained from natural populations in Kermanshah and Isfahan provinces, Iran, and (ii) five geographically distinct accessions acquired from the IPK Genebank (Gatersleben, Germany), consisting of CONI 4 (France), CONI 5 (Georgia), CONI 10 (Russia), CONI 17, and CONI 19 (Italy). All seed lots were labelled as publicly available and unrestricted. To initiate germination, seeds were placed on moistened cellulose pads, sealed in polyethylene bags, and subjected to cold stratification at approximately 5°C under dark conditions for two weeks. Following stratification, radicles measuring approximately 1.5–2 cm in length were selected for further anatomical investigation. For each accession, 5–10 seedlings were examined (typically 8–10), yielding a total of approximately 50 seedlings analysed across at least two independent germination batches per accession. In all accessions and independent germinations, structurally similar stomata were observed on the primary roots. After finding stomata in *C. maculatum*, we checked 31 additional Apiaceae species from 16 genera (Table 1), mostly from Apioideae, to see if root stomata are a trait more widespread in the subfamily. For these taxa, 2–3 seedlings per species were germinated and screened using the same germination protocol as above. Voucher specimens of wild populations were deposited in the Herbarium of the University of Isfahan (HUI), and voucher details of *C. maculatum* and the other Apiaceae screened species are provided in Supplementary Table S1.

**Table 1:** Species of Apiaceae examined for the presence of root stomata during seedling germination.

Genus	Species	Root stomata observed
*Ammi*	*A. majus*	−
*Anisotaenia*	*A. subvelutina*	−
*Bifora*	*B. testiculata*	−
*Conium*	*C. maculatum*	+ (consistent, this study)
*Cuminum*	*C. cyminum, C. setifolium*	−
*Demavendia*	*D. pastinacifolia*	−
*Dichoropetalum*	*D. paucijugum*	−
*Elwendia*	*E. afghanica, E. cylindrica, E. persica*	−
*Ferula*	*F. assa-foetida, F. cupularis, F. flabelliloba, F. gummosa, F. stenocarpa, F. szowitsiana*	−
*Foeniculum*	*F. vulgare*	−
*Petroselinum*	*P. crispum*	± (rare, nonreproducible)
*Pimpinella*	*P. anisum, P. leptoclada*	−
*Prangos*	*P. acaulis, P. calligonoides, P. crossoptera, P. ferulacea, P. gaubae*	± (rare, only *P. gaubae*)
*Scandix*	*S. pecten-veneris, S. stellata*	−
*Trachyspermum*	*T. ammi*	−
*Zeravschania*	*Z. aucheri, Z. pauciradiata*	−
*Zosima*	*Z. absinthifolia*	−

‘+’ = consistently present (only in *Conium maculatum*), ‘−’ = not detected, ‘±’ = extremely rare and nonreproducible (only in *Petroselinum crispum* and *Prangos gaubae*).

### Fixation and microscopy

Primary roots were fixed in a 3:1 (v/v) ethanol–glacial acetic acid solution for 24 h at room temperature. Following fixation, samples were stained with diluted methyl blue (∼1 min). In some preparations, carmine alum was applied as a secondary stain; however, its intensity was variable and not consistently visible in all sections. Excess stain was removed by rinsing in distilled water prior to microscopic examination. Microscopic preparations were obtained from the primary root. In a subset of seedlings, epidermal peels were briefly exposed (∼2 min) to Lugol’s iodine (I₂/KI) to test qualitatively for the presence of starch in guard cells and adjacent epidermal tissues. This treatment was performed on approximately 10 seedlings, with two independent repetitions per seedling. Excess stain was removed through an additional distilled water rinse. Epidermal peels or gentle surface squashes were used to obtain preparations of the root surface. Temporary mounts were prepared in distilled water and examined with a bright-field light microscope [Olympus BX40 light microscope (Olympus Corporation, Tokyo, Japan; https://www.olympus-lifescience.com)]. Stomatal structures were identified by standard anatomical criteria (paired reniform guard cells and a discernible pore). Images were acquired with a calibrated camera system. For the additional Apiaceae species listed in Table 1, stomata were recorded whenever present using the same staining and mounting procedures, but only a few seedlings per taxon were checked as a preliminary survey, using the same germination and anatomical protocol. Quantitative measurements of stomatal dimensions were performed on light micrographs using the software DigiMizer ver. 6.4.0. For each accession, stomatal length and width were measured from three to five micrographs selected from different seedlings. Mean values and standard errors were calculated for each accession.

### Transverse sectioning and staining

Transverse sections were obtained from the primary roots of *C. maculatum* using a double-edged razor blade. For each accession, multiple transverse sections from several seedlings were examined to ensure reproducibility. In total, transverse sections from all accessions were evaluated. Sections were mounted in distilled water (or 50% glycerol) for light microscopy (LM). Images were captured with an Olympus BX40 microscope, and scale bars were calibrated using a stage micrometre.

### Molecular identification

To verify species identity, molecular analyses were conducted on a representative seedling collected from a natural population. Total genomic DNA was extracted from young leaf tissue using a modified protocol based on the QIAGEN DNeasy Plant Mini Kit. Two widely accepted barcoding regions were amplified: the nuclear ribosomal internal transcribed spacer region (ITS) and the plastid *rps16* intron. Polymerase chain reaction was performed using primer sets ITSA/ITSB for ITS (Blattner 1999 ) and rps16F/rps16R for *rps16* (Shaw et al. 2007), following standard thermal cycling protocols. Amplified products were sequenced by a commercial provider, and sequence identity was determined through BLASTn (Camacho et al. 2009) searches against the NCBI GenBank database. Both ITS and *rps16* sequences matched GenBank entries of *C. maculatum* with 100% identity. These results supported the morphological identification based on diagnostic characters described in Flora Iranica (Hedge and Lamond 1987) and Flora of Iran (Mozaffarian 2007). The obtained sequences have been submitted to GenBank under accession numbers PX779193 (ITS) and PX781773 (*rps16*).

## Results

**Figure 1:**
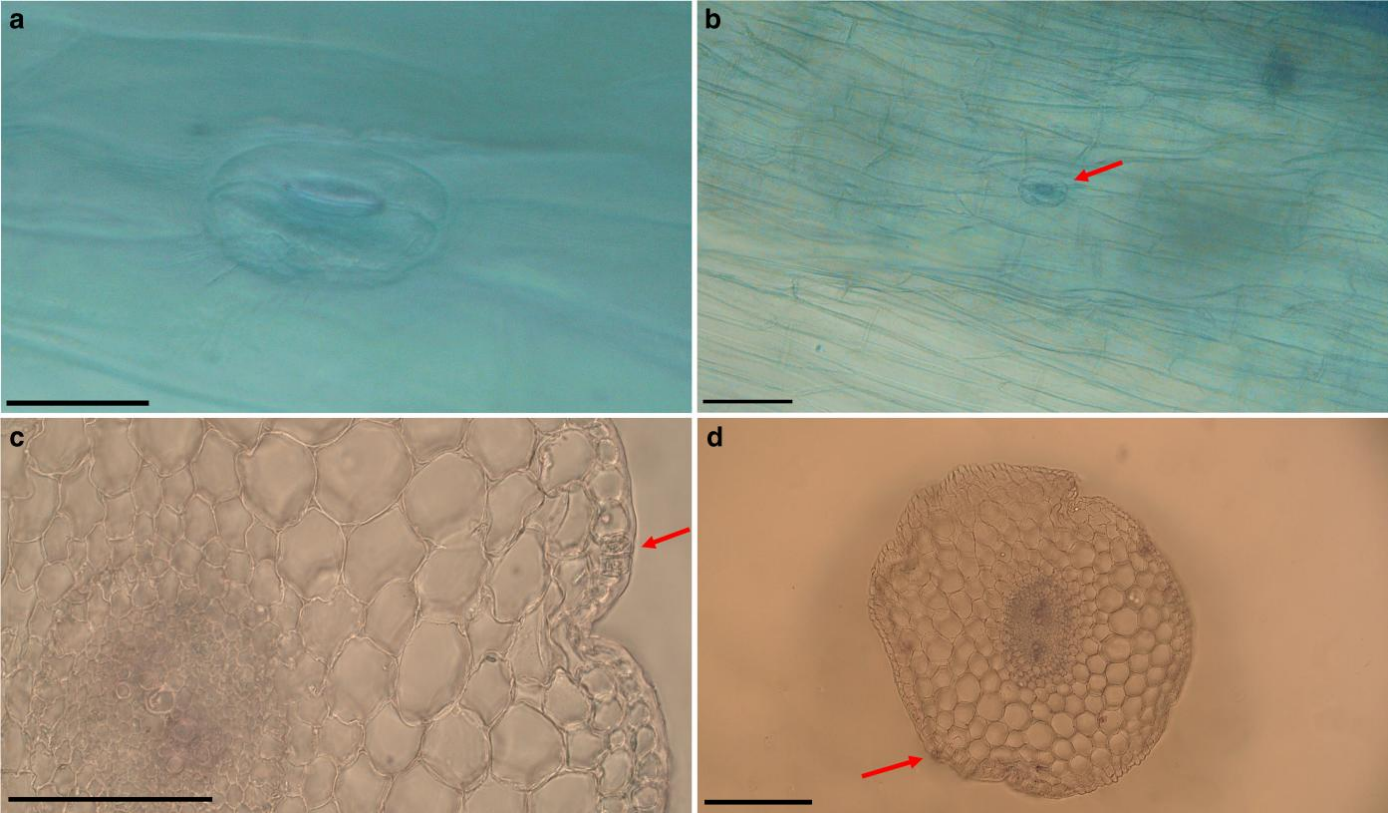
Light micrographs of primary root epidermis and transverse sections in *Conium maculatum*. (a) Stoma with paired guard cells. (b) Overview of root epidermis showing a stomatal complex (arrow). (c) Detail of a transverse section with a stomatal complex in the epidermis (arrow). (d) Whole transverse section of primary root, confirming root anatomy with a stomatal complex in the epidermis (arrow). Scale bars: a = 20 μm, b = 60 μm, c = 30 μm, d = 60 μm.

Microscopic observations confirmed the presence of stomata on the primary roots of *Conium maculatum* across all examined samples, including individuals from two wild-collected populations in Iran and five geographically diverse accessions obtained from the IPK Genebank. For each accession, multiple seedlings were analysed, and stomatal structures were detected in all cases. Stomata were observed on the primary roots and showed a sparse and irregular distribution. Quantitative measurements showed that mean stomatal length ranged from 29.09 to 45.18 µm among accessions, whereas mean stomatal width ranged from 19.63 to 25.12 µm (Supplementary Table S2). The length/width ratio varied between 1.16 and 2.10 across accessions. Stomata were observed in some preparations, but curvature of the root surface and mucilage residues frequently reduced image clarity. Detailed anatomical characterization was therefore conducted primarily through LM, which yielded clearer and more reproducible images. Under LM, stomata were embedded within the epidermal layer and exhibited reniform guard cells surrounding a central pore. No subsidiary cells were identified, indicating an anomocytic stomatal type (Fig. 1). In surface views, substomatal cavities were generally absent or inconspicuous. In transverse sections, a shallow substomatal space was occasionally observed directly beneath some stomata (Fig. 1; Supplementary Figure S1). The examined sections exhibited anatomical features consistent with a root structure, including a uniseriate epidermis and a broad parenchymatous cortex. These observations support the interpretation that the stomata occur on the primary root. Chloroplasts were not evident within guard cells under bright-field LM. After brief exposure (∼2 min) to I₂/KI, stomatal outlines became optically enhanced, and dark-stained granules were visible within guard cells and neighbouring epidermal cells, likely corresponding to starch-containing plastids. No qualitative differences in stomatal morphology or pattern were observed among samples of different geographic origins. No stomatal structures were observed on the root epidermis of the other Apiaceae taxa screened, except for *Prangos gaubae* and *Petroselinum crispum*, in which a putative stomatal complex was occasionally detected; however, these observations were not reproducible in additional individuals of the same species and therefore remain inconclusive.

## Discussion

In vascular plants, the root epidermis typically lacks stomata, a condition interpreted as a stable anatomical feature associated with subterranean growth and the absence of photosynthetic activity (Esau 1965, Raven 1977). Previous reports of stomata on primary roots have been limited to a few dicotyledonous taxa and generally interpreted as rare, developmentally transient, or environmentally induced phenomena. In *Helianthus annuus*, stomatal structures observed under moist-chamber conditions were misoriented and incompletely developed, suggesting an abnormal origin (Tietz and Urbasch 1977). In *Pisum sativum*, thick-walled stomata confined to the root apex were regarded as residual embryonic elements (Lefebvre 1985), whereas in *Pisum arvense* and *Ornithopus sativus*, irregular complexes in the elongation zone were short-lived and unresponsive to abscisic acid (Tarkowská and Wacowska 1988). A more persistent expression was described in a narrow apical root zone of *Ceratonia siliqua*, but the stomata lacked chloroplasts and did not show indicators of functional activity (Christodoulakis et al. 2002). Collectively, these studies have framed root stomata as highly exceptional structures lacking developmental stability across individuals or species. In contrast to these sporadic and often nonreproducible reports, the stomata observed in *Conium maculatum* were detected consistently across all examined seedlings, including two wild-collected Iranian populations and five geographically diverse genebank accessions. The stomata exhibited structurally complete guard cells of the anomocytic type. Their repeated detection under standard germination conditions, independent of seed source, indicates that the presence of stomata in *C. maculatum* does not represent an environmentally induced deviation or a transient embryonic remnant. Light-microscopic observations indicated that the stomata were embedded within the epidermal layer of the primary root. The examined anatomical features, including a uniseriate epidermis and a broad parenchymatous cortex, are consistent with a root identity for the tissues in which stomata were observed. These observations support the interpretation that the stomata occur on the primary root. Quantitative measurements indicated only minor variation in stomatal dimensions among accessions, supporting the interpretation of a consistent anatomical trait. A broader anatomical survey encompassing more than 30 additional Apiaceae species revealed no comparable stomatal structures on the primary root. Only *Prangos gaubae* and *Petroselium crispum* showed a single putative stomatal complex, but these observations were extremely infrequent and not reproducible in additional individuals, and therefore cannot be interpreted as evidence for root stomata in those taxa. These isolated occurrences may reflect developmental noise rather than a stable anatomical trait. Given the consistency of the observations in *C. maculatum*, the available evidence suggests that root stomata represent a species-level anatomical characteristic. However, conclusions regarding phylogenetic relevance remain premature. The genus *Conium* comprises several species distributed outside Iran, and additional sampling across the genus, as well as among closely related genera within tribe Scandiceae, will be necessary to determine whether this condition is unique to *C. maculatum* or is shared more broadly in the genus. The present findings expand the known range of epidermal developmental outcomes in roots by demonstrating that morphologically complete stomata can occur on a primary root within Apiaceae. This observation underscores the need for further comparative anatomical studies to evaluate whether latent developmental capacities for stomatal formation exist more broadly within the family. Our study establishes the first reproducible documentation of root stomata in Apiaceae and provides a foundation for future investigations into their developmental origin and taxonomic distribution.

## Conclusion

This study provides the first reproducible anatomical evidence of stomata on the root of *C. maculatum* within Apiaceae. Their occurrence was consistently observed across all examined accessions, including wild populations and diverse genebank materials, while comparable structures were absent or extremely rare in other surveyed taxa. These findings indicate that the presence of root stomata in *C. maculatum* represents a consistent species-level anatomical characteristic, though broader sampling across *Conium* and related genera will be necessary to determine its taxonomic extent. The results expand current knowledge of root epidermal anatomy in Apiaceae and highlight the value of targeted anatomical surveys for identifying unexpected developmental features within the family.

## Supplementary Material

plag001_Supplementary_Data

## Data Availability

All data underlying this study are included within the article and its online Supplementary material.

## References

[plag001-B1] Ajani Y, Ajani A, Cordes JM et al Phylogenetic analysis of nrDNA ITS sequences reveals relationships within five groups of Iranian Apiaceae subfamily Apioideae. Taxon 2008;57:383–401. 10.2307/25066011

[plag001-B2] Blattner FR . Direct amplification of the entire ITS region from poorly preserved plant material using recombinant PCR. Biotechniques 1999;27:1180–6. 10.2144/99276st04 10631497

[plag001-B100] Camacho C, Coulouris G, Avagyan V et al BLAST+: architecture and applications. BMC Bioinformatics 2009;10:421. 10.1186/1471-2105-10-421 20003500 PMC2803857

[plag001-B3] Christodoulakis NS, Menti J, Galatis B. Structure and development of stomata on the primary root of *Ceratonia siliqua* L. Ann Bot 2002;89:23–9. 10.1093/aob/mcf002 12096815 PMC4233769

[plag001-B4] Clarkson JJ, Zuntini AR, Maurin O et al A higher-level nuclear phylogenomic study of the carrot family (Apiaceae). Am J Bot 2021;108:1252–69. 10.1002/ajb2.1701 34287829

[plag001-B5] Downie SR, Spalik K, Katz-Downie DS et al Major clades within Apiaceae subfamily Apioideae as inferred by phylogenetic analysis of nrDNA ITS sequences. Plant Diversity and Evolution 2010;128:111–36. 10.1127/1869-6155/2010/0128-0005

[plag001-B6] Esau K . Plant Anatomy, 2nd edn. New York: Wiley, 1965.

[plag001-B7] Fahn A . Plant Anatomy, 3rd edn. Oxford: Pergamon Press, 1982.

[plag001-B8] Hedge IC, Lamond JM. Umbelliferae. In: Rechinger KH (ed.) Flora Iranica, Vol. 162. Graz: Akademische Druck- und Verlagsanstalt, 1987.

[plag001-B9] Lefebvre DD . Stomata on the primary root of *Pisum sativum* L. Ann Bot 1985;55:337–41. 10.1093/oxfordjournals.aob.a086910

[plag001-B10] Magee AR, Calviño CI, Liu M et al New tribal delimitations for the early-divergent lineages of Apiaceae subfamily Apioideae. Taxon 2010;59:567–80. 10.1002/tax.592021

[plag001-B11] Mousavi S, Mozaffarian V, Mummenhoff K et al An updated lineage-based tribal classification of Apiaceae subfamily Apioideae with special focus on Iranian genera. Syst Biodivers 2020;19:89–109. 10.1080/14772000.2020.1834002

[plag001-B12] Mozaffarian V . Flora of Iran: Umbelliferae, Vol. 54. Tehran: Research Institute of Forests and Rangelands Publications, 2007.

[plag001-B13] Raven PH . Biology of Plants, 2nd edn. New York: Worth Publishers, 1977.

[plag001-B14] Shaw J, Lickey EB, Schilling EE et al Comparison of whole chloroplast genome sequences to choose noncoding regions for phylogenetic studies in angiosperms: the tortoise and the hare III. Am J Bot 2007;94:275–88. 10.3732/ajb.94.3.275 21636401

[plag001-B15] Tarkowska JA, Wacowska M. The significance of the presence of stomata on seedling roots. Ann Bot 1988;61:305–10. 10.1093/oxfordjournals.aob.a087558

[plag001-B16] Tietz H, Urbasch I. Spaltöffnungen an der Keimwurzel von *Helianthus annuus* L. Naturwissenschaften 1977;64:533. 10.1007/BF00483558927537

