# Supplementary material for: Root stomata in Conium maculatum (Apiaceae): anatomically verified occurrence and a comparative survey across Apioideae
Source: AoB Plants. 2026 Feb 10;18(1):plag001. doi: 10.1093/aobpla/plag001 (PMC12888389; doi:10.1093/aobpla/plag001)
Supplement: plag001_Supplementary_Data [file plag001_supplementary_data.zip › Table S1.docx]

**Supplementary Table S1.** Voucher specimens of Apiaceae species examined for the presence of root stomata. All specimens are deposited in the Herbarium of the University of Isfahan (HUI).

| No. | Taxon | Locality | Herbarium Number |
| --- | --- | --- | --- |
| 1 | *Ammi majus* L. | Lorestan | 26199 |
| 2 | *Anisotaenia subvelutina* (Rech.f.) Lyskov & Bagheri (Syn: *Ferulago subvelutina* Rech.f.) | Isfahan | 23005 |
| 3 | *Bifora testiculata* (L.) Spreng. | Lorestan | 26185 |
| 4 | *Conium maculatum* L. | Isfahan | 26206 |
| 5 | *Conium maculatum* L. | Kermanshah | 26207 |
| 6 | *Cuminum cyminum* L. | Isfahan | 26204 |
| 7 | *Cuminum setifolium* (Boiss.) Koso-Pol. | Razavi Khorasan | 26187 |
| 8 | *Demavendia pastinacifolia* (Boiss. & Hausskn.) Pimenov | Isfahan | 23721 |
| 9 | *Dichoropetalum paucijugum* (Pimenov) Pimenov & Kljuykov (Syn: *Johrenia paucijuga*) | Lorestan | 26197 |
| 10 | *Elwendia afghanica* (Beauverd) Pimenov & Kljuykov | Razavi Khorasan | 26188 |
| 11 | *Elwendia cylindrica* (Boiss. & Hausskn.) Pimenov & Kljuykov (Syn: *Bunium cylindricum*) | Isfahan | 26200 |
| 12 | *Elwendia persica* (Boiss.) Pimenov & Kljuykov (Syn: *Bunium persicum*) | Isfahan | 23723 |
| 13 | *Ferula assa-foetida* L. | Isfahan | 24038 |
| 14 | *Ferula cupularis* (Boiss.) Spalik & S.R.Downie (Syn: *Leutea cupularis* (Boiss.) Pimenov) | Isfahan | 26205 |
| 15 | *Ferula flabelliloba* Rech.f. & Aellen | Razavi Khorasan | 26189 |
| 16 | *Ferula gummosa* Boiss. | Tehran | 26201 |
| 17 | *Ferula stenocarpa* Boiss. & Hausskn. | Fars | 24039 |
| 18 | *Ferula szowitsiana* DC. | Razavi Khorasan | 26190 |
| 19 | *Foeniculum vulgare* Mill. | Razavi Khorasan | 26191 |
| 20 | *Petroselinum crispum*(Mill.) Fuss | Azarbaijan | 26208 |
| 21 | *Pimpinella anisum* L. | Tehran | 26203 |
| 22 | *Pimpinella leptoclada* (Aitch. & Hemsl.) Mousavi, Mozaff. & Zarre (Syn: *Aphanopleura leptoclada*) | Razavi Khorasan | 26192 |
| 23 | *Prangos acaulis* (DC.) Bornm. (Syn: *Cachrys acaulis*) | Zanjan | 26202 |
| 24 | *Prangos calligonoides* Rech.f. | Isfahan | 26155 |
| 25 | *Prangos crossoptera* Herrnst. & Heyn | Kurdistan | 26156 |
| 26 | *Prangos ferulacea* (L.) Lindl. | Kurdistan | 26161 |
| 27 | *Prangos gaubae* (Bornm.) Herrnst. & Heyn | Tehran | 26162 |
| 28 | *Scandix pecten-veneris* L. | Lorestan | 26193 |
| 29 | *Scandix stellata* Banks & Sol. | Lorestan | 26194 |
| 30 | *Trachyspermum ammi* (L.) Sprague (Syn: *Carum copticum*) | Razavi Khorasan | 26195 |
| 31 | *Zeravschania aucheri* (Boiss.) Pimenov | Razavi Khorasan | 26196 |
| 32 | *Zeravschania pauciradiata*(Tamamsch.) Pimenov  (Syn: *Johreniopsis oligactis*) | Lorestan | 26198 |
| 33 | *Zosima absinthifolia* (Vent.) Link | Isfahan | 23736 |
