# Supplementary material for: Root stomata in Conium maculatum (Apiaceae): anatomically verified occurrence and a comparative survey across Apioideae
Source: AoB Plants. 2026 Feb 10;18(1):plag001. doi: 10.1093/aobpla/plag001 (PMC12888389; doi:10.1093/aobpla/plag001)
Supplement: plag001_Supplementary_Data [file plag001_supplementary_data.zip › Table S2.docx]

**Supplementary Table S2.** Quantitative measurements of stomatal length and width, and the length/width ratio (L/W) for each accession of *Conium maculatum*. Data are presented as mean ± standard error (SE). “n” indicates the number of measured micrographs per accession.

| **Accessions** | **Sample Size (n)** | **Mean Stomatal Length (µm)** | **Mean Stomatal Width (µm)** | **L/W** |
| --- | --- | --- | --- | --- |
| CONI 4 (France) | 5 | 41.79 ± 2.32 | 23.63 ± 1.27 | 1.76 |
| CONI 5 (Georgia) | 5 | 41.31 ± 2.10 | 19.63 ± 0.98 | 2.10 |
| CONI 10 (Russia) | 3 | 44.90 ± 5.29 | 22.83 ± 0.65 | 1.96 |
| CONI 17 (Italy) | 4 | 36.53 ± 1.61 | 21.98 ± 0.37 | 1.66 |
| CONI 19 (Italy) | 4 | 29.09 ± 1.31 | 25.12 ± 1.24 | 1.15 |
| Kermanshah (Iran) | 3 | 45.17 ± 1.60 | 23.17 ± 0.98 | 1.94 |
| Isfahan (Iran) | 5 | 34.51 ± 1.50 | 21.62 ± 0.69 | 1.59 |
