# Supplementary figures and images for: Root stomata in Conium maculatum (Apiaceae): anatomically verified occurrence and a comparative survey across Apioideae
Source: AoB Plants. 2026 Feb 10;18(1):plag001. doi: 10.1093/aobpla/plag001 (PMC12888389; doi:10.1093/aobpla/plag001)

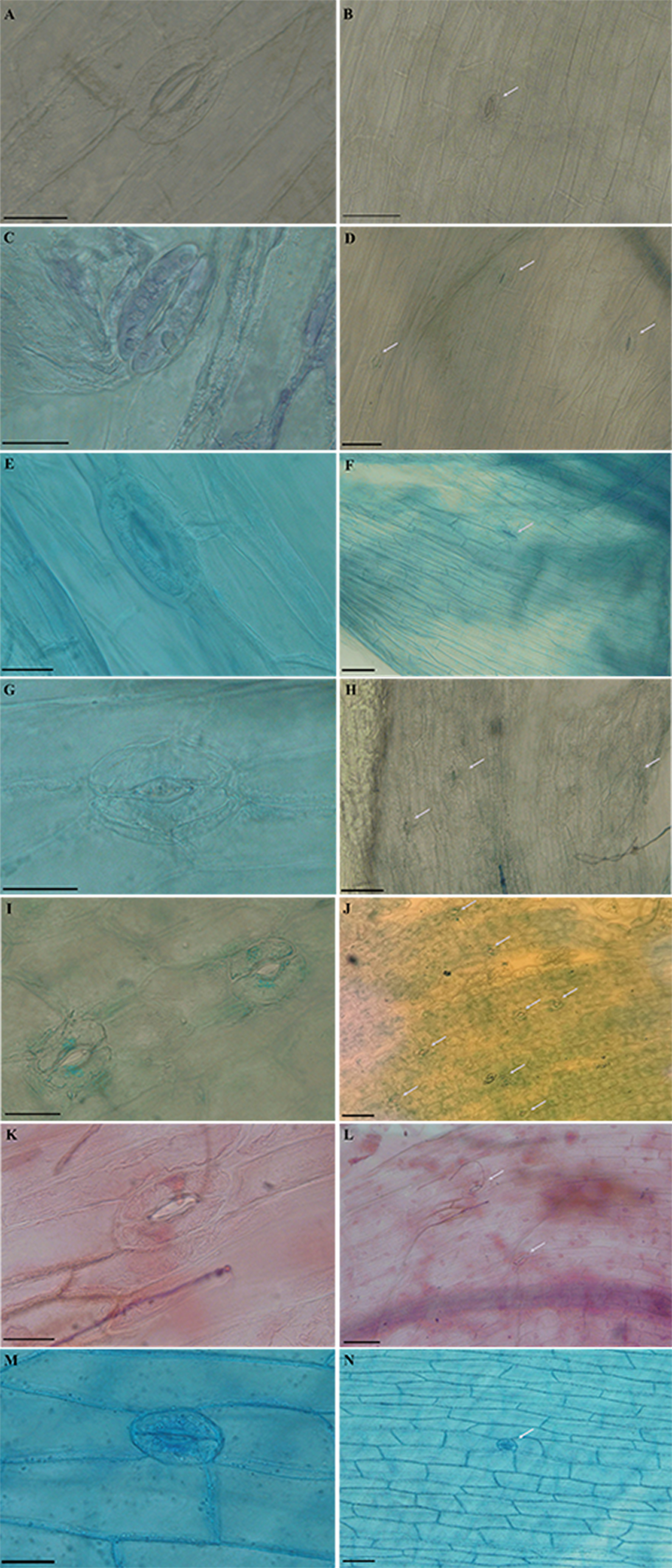

Supplement: plag001_Supplementary_Data [file plag001_supplementary_data.zip › Fig S1.jpg]
